# Metabolic traits affecting the relationship between liver fat and intrapancreatic fat: a mediation analysis

**DOI:** 10.1007/s00125-022-05793-4

**Published:** 2022-10-04

**Authors:** Juyeon Ko, Ivana R. Sequeira, Loren Skudder-Hill, Jaelim Cho, Sally D. Poppitt, Maxim S. Petrov

**Affiliations:** 1 School of Medicine, University of Auckland, Auckland, New Zealand; 2 Human Nutrition Unit, School of Biological Sciences, University of Auckland, Auckland, New Zealand; 3 High Value Nutrition, National Science Challenge, Auckland, New Zealand; 4 Riddet Centre of Research Excellence for Food and Nutrition, Palmerston North, New Zealand

**Keywords:** Glucose metabolism, Incretins, Intrapancreatic fat, Lipids, Liver enzymes, Liver fat, Mediation, Pancreatic hormones

## Abstract

**Aims/hypothesis:**

The clinical importance of fat deposition in the liver and pancreas is increasingly recognised. However, to what extent deposition of fat in these two depots is affected by intermediate variables is unknown. The aim of this work was to conduct a mediation analysis with a view to uncovering the metabolic traits that underlie the relationship between liver fat and intrapancreatic fat deposition (IPFD) and quantifying their effect.

**Methods:**

All participants underwent MRI/magnetic resonance spectroscopy on the same 3.0 T scanner to determine liver fat and IPFD. IPFD of all participants was quantified manually by two independent raters in duplicate. A total of 16 metabolic traits (representing markers of glucose metabolism, incretins, lipid panel, liver enzymes, pancreatic hormones and their derivatives) were measured in blood. Mediation analysis was conducted, taking into account age, sex, ethnicity and BMI. Significance of mediation was tested by computing bias-corrected bootstrap CIs with 5000 repetitions.

**Results:**

A total of 353 individuals were studied. Plasma glucose, HDL-cholesterol and triacylglycerol mediated 6.8%, 17.9% and 24.3%, respectively, of the association between liver fat and IPFD. Total cholesterol, LDL-cholesterol, alanine aminotransferase, aspartate aminotransferase, alkaline phosphatase, γ-glutamyl transpeptidase, insulin, glucagon, amylin, C-peptide, HbA_1c_, glucagon-like peptide-1 and gastric inhibitory peptide did not mediate the association between liver fat and IPFD.

**Conclusions/interpretation:**

At least one-quarter of the association between liver fat and IPFD is mediated by specific blood biomarkers (triacylglycerol, HDL-cholesterol and glucose), after accounting for potential confounding by age, sex, ethnicity and BMI. This unveils the complexity of the association between the two fat depots and presents specific targets for intervention.

**Graphical abstract:**

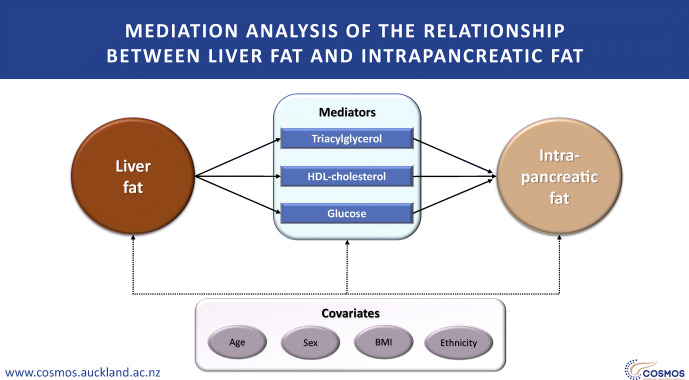

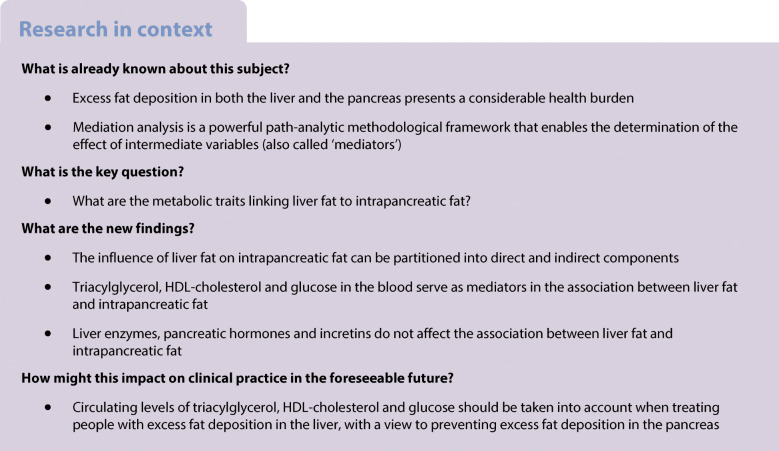



## Introduction

While the dangers of excess body fat in general became well appreciated in the 20th century, the risks associated with excess ectopic fat deposition have been progressively brought to the fore in the 21st century. In particular, excess deposition of fat in the parenchymal cells of the liver (fatty liver disease) has emerged as the most common disorder of the liver in the western world and eastern world alike [1–5]. This fat deposition is a growing cause of cirrhosis, hepatocellular cancer and end-stage liver disease, which may require liver transplantation. Fatty liver disease is also a risk factor for CVD (regardless of traditional risk factors such as arterial hypertension) and chronic kidney disease [5]. Another parenchymal organ often bedevilled by excess fat deposition is the pancreas. Fatty pancreas disease is the most common disorder of the pancreas and is a harbinger of pancreatitis and pancreatic cancer [6–8]. Pancreatic cancer is one of the most lethal diseases, with the mortality rate being similar to incidence (seven and eight cases per 100,000 person-years, respectively) [9]. Pancreatitis has an incidence rate of 43 cases per 100,000 person-years and, while mortality from this disease is relatively low, can result in numerous new-onset metabolic sequelae such as post-pancreatitis diabetes mellitus, exocrine pancreatic dysfunction and osteopathy [9–12]. The burden of both pancreatic cancer and pancreatitis is projected to increase substantially by 2050 [13]. The liver and the pancreas share a common developmental origin and numerous studies over the past decade have shown a significant association between liver fat and intrapancreatic fat deposition (IPFD) [14]. While early studies on the topic should be interpreted with caution because of their use of ultrasound (which is semi-quantitative and operator-dependent) and small sample size [15–17], the association between liver fat and IPFD was conclusively shown in a large 2014 population-based study that employed chemical shift-encoded MRI (the gold standard for quantifying IPFD non-invasively) [18].

Now that scientific knowledge in the area is no longer in its rudimentary stage, it behoves researchers to develop a fine-grained understanding of the mechanisms that underlie the link between liver fat and IPFD. While BMI and sex are obvious confounders of the association between the two entities, the epistemological challenge is to establish exactly how these entities become intertwined [19]. Mediation analysis is positioned well to test hypotheses about the mechanisms that are at work as it determines the extent to which a potential causal variable influences an outcome, through an intermediate variable (also called ‘mediator’). Specifically, this path-analytic methodological framework enables partitioning of the influence of liver fat on IPFD into indirect (i.e. through the mediator of interest) and direct (i.e. through other mechanisms) components and allows the quantification of both (i.e. estimating the proportion mediated) [20]. As with any causal inference method, mediation analysis requires assumptions to be made about the causality of the effects in the mediation model. Specifically, it is assumed that changes in liver fat cause changes in metabolic traits and that changes in metabolic traits cause changes in intrapancreatic fat. The biological plausibility of these assumptions is strong in regard to liver enzymes, lipid panel, markers of glucose metabolism, incretins and pancreatic hormones. Fatty liver disease is widely regarded as the most common cause of elevated liver enzymes [2, 5, 21]. Liver enzymes were significantly associated with IPFD in our 2017 meta-analysis of biomarkers of IPFD (encompassing nearly 12,000 individuals from 17 observational studies) that informed the design of the present study [22]. Notably, γ-glutamyl transferase had the strongest association of all the biomarkers studied (though it was investigated in two studies only). Lipid metabolism is dysregulated in fatty liver disease as increased liver fat results in hepatic overproduction of VLDL particles and dysregulated clearance of lipoproteins from the circulation [1, 23]. Lipid panel was significantly associated with IPFD in the above-mentioned 2017 meta-analysis, with triacylglycerol being the most frequently investigated biomarker [22]. A Mendelian randomisation study demonstrated that liver fat causes type 2 diabetes (defined based on HbA_1c_ and/or fasting plasma glucose levels) [24]. Hyperglycaemia in the non-diabetic range (defined based on HbA_1c_ and/or fasting plasma glucose levels) was an independent predictor of increased IPFD after 5 years of follow-up in a longitudinal cohort study of individuals without diabetes [25]. Liver fat is almost universally associated with insulin resistance, with resulting changes in secretion of both pancreatic hormones and incretins [5, 26]. Pancreatic hormones were significantly associated with IPFD (with no heterogeneity between the studies that investigated insulin) in the above-mentioned 2017 meta-analysis [22].

The present study aimed to disentangle the relationships between the above metabolic traits and liver fat and IPFD, using mediation analysis.

## Methods

### Study design

This cross-sectional study, with the enrolment of four individual cohorts [27], was conducted at the University of Auckland (Auckland, New Zealand) and received ethical approval by the New Zealand Health and Disability Ethics Committee (13/STH/182, 16/STH/23, 17/NTA/172, 18/NTB/1). People aged >18 years residing in Auckland were recruited following written informed consent. They had no personal history of acute infectious or inflammatory disorders requiring medical treatment or evaluation in the preceding 6 months. Individuals were excluded if they had participated in a weight-loss programme, received dietetic support or education, undergone liver, pancreatic or bariatric surgery or organ transplantation, had chronic pancreatitis or any other pathology of the pancreas detectable on cross-sectional imaging, received any radiological or endoscopic intervention involving the liver or the pancreas, had malignancy, chronic liver disease or autoimmune disease, used systemic corticosteroids, had health issues that preclude undergoing MRI (e.g. end-stage renal failure, congestive heart failure, mental disorders), had metallic implants, heart pacemakers or other implantable electronic devices, or were pregnant or breastfeeding.

### MRI measurements

#### Imaging protocol

A single 3.0 Tesla MAGNETOM Skyra scanner, VE 11A (Siemens, Erlangen, Germany) at the Centre for Advanced Magnetic Resonance Imaging at the University of Auckland (Auckland, New Zealand) was used to acquire abdominal images using the same protocol for all study participants. The protocol involved participants lying in the supine position, holding their breath for 11 s at end-expiration. Axial longitudinal relaxation time (T1)-weighted volumetric interpolated breath-hold examination Dixon sequence was applied with the following parameters: true form abdomen shim mode; field of view (FOV), 440 mm; basic resolution, 512; echo time (TE), 2.46 ms, 3.69 ms; repetition time (TR), 5.82 ms; flip angle, 9°; pixel bandwidth, 750 Hz; signal average, 1; slice thickness, 5 mm; field of view, 500 × 400 mm; matrix, 512 × 410; and partial Fourier and parallel imaging with a total acceleration factor of 2.8. Four types of images were generated: in-phase; out-of-phase; fat only; and water only.

#### Liver fat

Magnetic resonance spectroscopy (MRS) was used to determine liver fat. A single voxel (20 mm × 20 mm × 20 mm) was placed in the right lobe of the liver, away from the blood vessels and bile ducts and at least 10 mm away from the organ’s edge. Automated shimming was performed prior to signal acquisition to improve the homogeneity of B0, the main static magnetic field. Spectra were acquired using a free-breathing navigator-triggered spin-echo acquisition with the following parameters: TE, 33 ms; TR, 3000 ms; 50 averages. The acquisition time for each spectrum was 5 min. Both water-suppressed and non-water-suppressed spectra were taken, with the non-water-suppressed spectrum being the reference for liver fat quantification. Spectra were processed and analysed using the SIVIC software (University of California-San Francisco, USA). The fat fraction (%) was defined as the area under fat peak divided by area under fat and water peaks multiplied by 100 [28].

#### Intrapancreatic fat

IPFD was measured manually using a modified ‘MR-opsy’ technique, described in detail elsewhere [29]. Briefly, two candidate slices with clear visualisation of the pancreas were selected from a series of abdominal scans. Three regions of interest were placed in the head, body and tail regions of the pancreas to estimate IPFD. A thresholding range of 1–20% was applied to prevent the potential inclusion of non-parenchymal tissues (such as visceral fat, the main pancreatic duct, blood vessels) within the selected regions of interest [30]. IPFD was calculated, using ImageJ software (National Institutes of Health, USA), as the average pancreatic fat fraction of the two slices. Two raters measured IPFD independently for each participant and average values of two measurements were used for statistical analyses. Intra-class correlation was calculated to assess the inter-rater reliability. The inter-rater reliability was considered excellent if intra-class correlation was more than 0.90 [31].

### Laboratory measurements

Venous blood samples were obtained from each participant at the time of their participation, after at least 8 h of fasting. These blood samples were centrifuged at 4000 *g* (4°C); plasma and serum were separated into aliquots and stored at −80°C until further use. Laboratory measurements were performed separately for each of the four individual cohorts, using the same laboratory methods. HbA_1c_ was measured using a chromatography assay. Fasting plasma glucose was measured using a hexokinase colorimetric assay. Liver enzymes and lipid panel were analysed using standard methods in LabPlus, the tertiary referral medical laboratory of Auckland City Hospital (Auckland, New Zealand). Pancreatic hormones (insulin, glucagon, amylin [islet amyloid polypeptide]), their derivatives (C-peptide), and incretins (gastric inhibitory peptide [GIP], total glucagon-like peptide-1 [GLP-1]) were measured using the MILLIPLEX MAP human metabolic hormone magnetic bead panel based on the Luminex xMAP technology (Merck, Hesse, Germany) in line with the manufacturer’s instructions. Results were quantified based on fluorescent reporter signals recorded by the Luminex xPONENT software (MILLIPLEX Analyst 5.1). The intra-assay and inter-assay CV for all analytes was <10% and <15%, respectively.

### Covariates

Anthropometric data (height and weight) of all participants were recorded to calculate BMI. All measurements were taken with participants wearing light clothing, and height and weight were measured in a standing position without shoes or headgear. Ethnicity was categorised as European White, Asian and Others.

### Statistical analysis

Analyses were conducted using SPSS version 27.0 (IBM Corp., NY, USA) and SAS version 9.4 for Windows (SAS Institute, USA). Data were expressed as median and IQR or frequency. Data with skewed distribution were log-transformed when appropriate. A single-mediator model with single-level data for the mediation analysis was built to investigate whether the studied metabolic traits are mediators in the association between liver fat and IPFD [32]. Age, sex, ethnicity and BMI were treated as confounders. PROCESS macro for mediation analysis (https://processmacro.org/ version 3.4.1) was used to test statistical significance and magnitude of mediation in ordinary least-squares regression models, according to the method of Preacher and Hayes [33]. First, we calculated direct effect estimates of liver fat on IPFD (which included the exposure, confounders and mediator as independent variables). Then, we estimated the effect of liver fat on individual potential mediators. All the studied metabolic traits were considered as potential mediators. The indirect effect of potential mediators was then calculated by computing the product of the two regression coefficients of the potential mediators on liver fat and IPFD. The magnitude of indirect effect was calculated by dividing the coefficient of the indirect effect by the coefficient of the direct effect, according to the method of Preacher and Hayes [33].

Significance of mediation was tested by computing bias-corrected bootstrap CIs. Bootstrapping (a non-parametric resampling procedure) was used because it did not impose the assumption of normality of the sampling distribution. Bootstrapping involved repeated sampling from the dataset to estimate the indirect effect in each resampled dataset. By repeating this process 5000 times, an empirical approximation of the sampling distribution of the quantified indirect effect of the independent variable on the dependent variable through each potential mediator was built and used to yield CIs for the indirect effect [34]. A two-sided *p* value of less than 0.05 was deemed statistically significant. The mediation analysis was reported in line with the AGReMA (A Guideline for Reporting Mediation Analyses) guidelines [35].

## Results

### Characteristics of participants

**Fig. 1:**
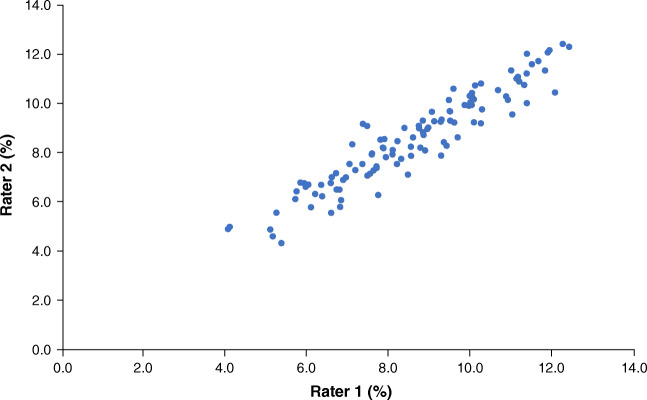
Concordance between intrapancreatic fat measurements by two independent raters

A total of 410 participants met the inclusion criteria. Participants who had pathology of the pancreas on cross-sectional imaging (*n*=22), major health problems that precluded undergoing MRI (*n*=8), malignancy (*n*=3), autoimmune disease (*n*=3), an organ transplant (*n*=1), a heart pacemaker (*n*=1), or who used systemic corticosteroids (*n*=1) were excluded. In addition, participants were excluded if MRS was abandoned (*n*=15), the pancreas was not visible in its entirety on MRI (*n*=1), the participant developed claustrophobia while in the scanner (*n*=1), or i.v. cannulation failed (*n*=1). Three hundred and fifty-three individuals were analysed. Their median (IQR) age was 50.0 (37.0–60.0) years, and 151 (42.8%) of the study participants were men. Other baseline characteristics are presented in Table 1. The median (IQR) liver fat and IPFD was 5.7 (3.1–12.0)% and 8.7 (7.0–10.1)%, respectively. The intra-class correlation of IPFD measurement was 0.967 (95% CI 0.960, 0.973) (Fig. 1).

**Table 1:** Characteristics of the study cohort

Characteristic	Measurement
No. of participants	353
Age, years	50.0 (37.0–60.0)
Men, *n* (%)	151 (42.8)
Ethnicity, *n* (%)	
European White	115 (32.6)
Asian	175 (49.6)
Other	63 (17.8)
BMI, kg/m^2^	26.8 (24.3–29.7)
HbA_1c_, mmol/mol	35.0 (32.0–39.0)
HbA_1c_, %	5.4 (5.1–5.7)
Fasting plasma glucose, mmol/l	5.3 (4.9–5.7)
Fasting insulin, pmol/l	35.9 (23.6–57.7)
Total amylin, pg/ml	30.7 (14.4–43.0)
C-peptide, nmol/l	0.1 (0.04–0.26)
Total GLP-1, pg/ml	149.3 (98.8–262.7)
GIP, pg/ml	67.9 (36.3–111.7)
Glucagon, ng/l	53.3 (36.5–85.3)
ALT, U/l	20.3 (12.9–29.9)
AST, U/l	21.7 (17.2–26.7)
ALP, U/l	76.5 (62.4–94.8)
GGT, U/l	20.2 (14.6–33.4)
Total cholesterol, mmol/l	4.8 (4.2–5.6)
HDL-cholesterol, mmol/l	1.3 (1.0–1.6)
LDL-cholesterol, mmol/l	2.7 (2.2–3.4)
Triacylglycerol, mmol/l	1.2 (0.9–1.8)

Data are presented as median (IQR) or *n* (%)

Data on HbA_1c_, fasting plasma glucose, fasting insulin, amylin, C-peptide, total GLP-1, GIP, glucagon, ALT, AST, ALP, GGT and triacylglycerol were log-transformed

ALP, alkaline phosphatase; ALT, alanine aminotransferase; AST, aspartate aminotransferase; GGT, γ-glutamyl transpeptidase

### Lipid panel as mediators

**Fig. 2:**
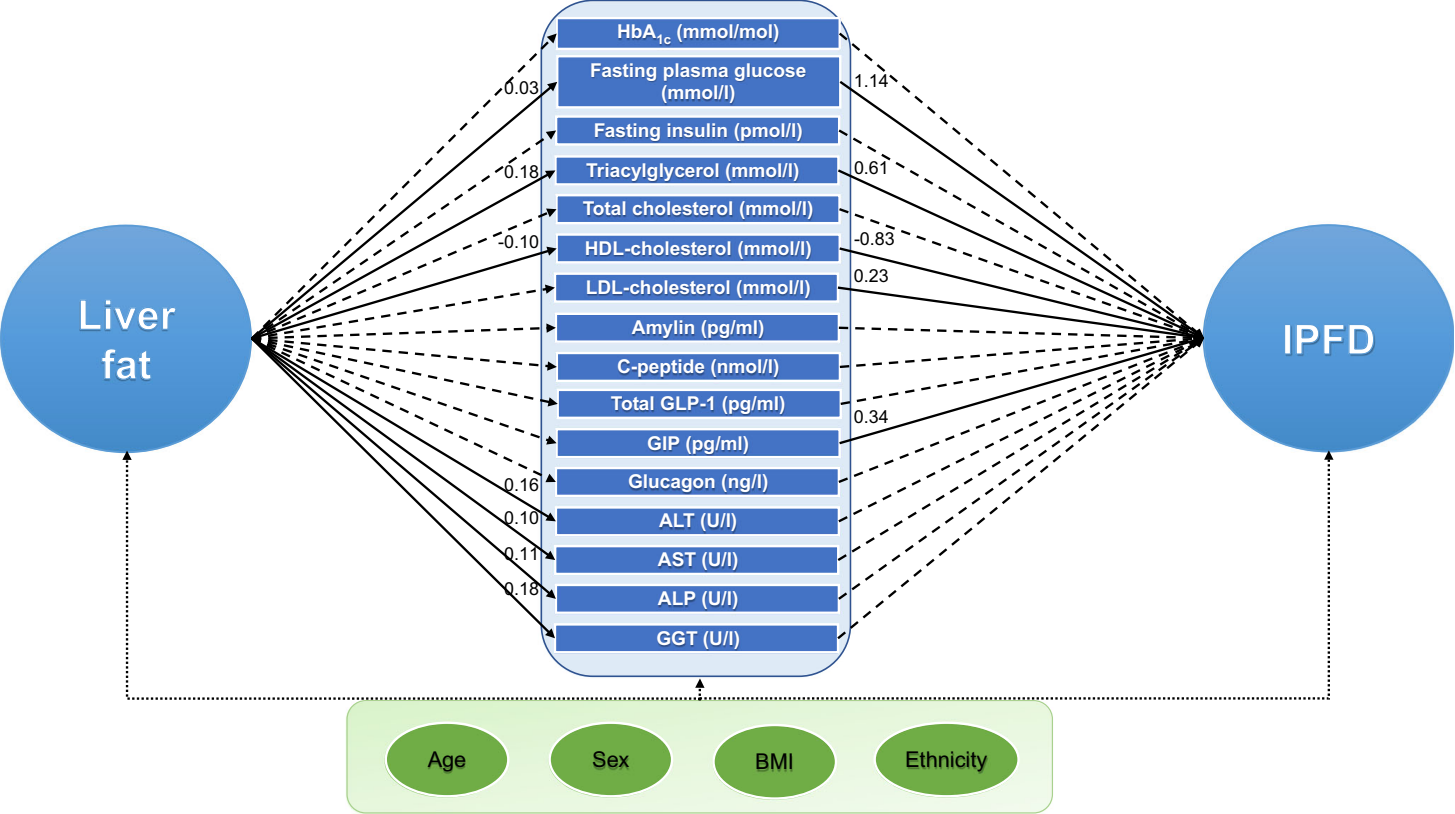
Scheme of the study design and key findings. Single-headed arrows represent regression paths, circles represent independent and dependent variables, ovals represent covariates and rectangles represent potential mediators. Standardised coefficients are shown for statistically significant indirect effects (solid lines) only. Dashed lines represent non-significant indirect effects; dotted lines represent covariates. ALP, alkaline phosphatase; ALT, alanine aminotransferase; AST, aspartate aminotransferase; GGT, γ-glutamyl transpeptidase

**Fig. 3:**
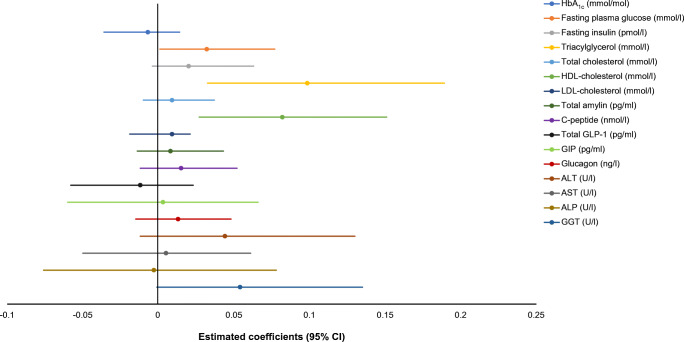
Indirect effects (with 95% CIs) from the bootstrap samples. ALP, alkaline phosphatase; ALT, alanine aminotransferase; AST, aspartate aminotransferase; GGT, γ-glutamyl transpeptidase

Triacylglycerol, total cholesterol, HDL-cholesterol and LDL-cholesterol were considered as potential mediators in the association between liver fat and IPFD (Fig. 2). While the exposure–mediator effect was statistically significant for triacylglycerol and HDL-cholesterol, the mediator–outcome effect was statistically significant for triacylglycerol, HDL-cholesterol and LDL-cholesterol (Table 2). Based on the statistical significance of the indirect effect estimates, triacylglycerol and HDL-cholesterol were mediators whereas total cholesterol and LDL-cholesterol were not (Fig. 3). Triacylglycerol and HDL-cholesterol mediated 24.3% and 17.9%, respectively, of the association between liver fat and IPFD (Table 3).

**Table 2:** Effects of liver fat on metabolic traits and of metabolic traits on intrapancreatic fat

Trait	Standard coefficient	SE	*p* value
Liver fat and metabolic traits			
Effect of liver fat on HbA_1c_	0.016	0.013	0.172
Effect of liver fat on fasting plasma glucose	0.028	0.011	0.008
Effect of liver fat on fasting insulin	0.083	0.051	0.105
Effect of liver fat on total cholesterol	0.073	0.070	0.297
Effect of liver fat on HDL-cholesterol	−0.100	0.253	<0.001
Effect of liver fat on LDL-cholesterol	0.038	0.060	0.527
Effect of liver fat on triacylglycerol	0.175	0.039	<0.001
Effect of liver fat on ALT	0.163	0.055	0.004
Effect of liver fat on AST	0.098	0.040	0.015
Effect of liver fat on ALP	0.108	0.032	0.001
Effect of liver fat on GGT	0.176	0.069	0.019
Effect of liver fat on total amylin	0.072	0.063	0.257
Effect of liver fat on C-peptide	0.132	0.103	0.204
Effect of liver fat on total GLP-1	−0.050	0.076	0.513
Effect of liver fat on GIP	0.009	0.089	0.920
Effect of liver fat on glucagon	0.050	0.058	0.394
Metabolic traits and intrapancreatic fat			
Effect of HbA_1c_ on intrapancreatic fat	−0.369	0.504	0.465
Effect of fasting plasma glucose on intrapancreatic fat	1.142	0.565	0.044
Effect of fasting insulin on intrapancreatic fat	0.243	0.137	0.077
Effect of total cholesterol on intrapancreatic fat	0.120	0.088	0.172
Effect of HDL-cholesterol on intrapancreatic fat	−0.832	0.238	0.001
Effect of LDL-cholesterol on intrapancreatic fat	0.230	0.106	0.031
Effect of triacylglycerol on intrapancreatic fat	0.614	0.205	0.003
Effect of ALT on intrapancreatic fat	0.270	0.203	0.187
Effect of AST on intrapancreatic fat	0.048	0.282	0.866
Effect of ALP on intrapancreatic fat	−0.025	0.349	0.943
Effect of GGT on intrapancreatic fat	0.209	0.161	0.057
Effect of total amylin on intrapancreatic fat	0.113	0.141	0.422
Effect of C-peptide on intrapancreatic fat	0.114	0.087	0.194
Effect of total GLP-1 on intrapancreatic fat	0.244	0.124	0.050
Effect of GIP on intrapancreatic fat	0.338	0.106	0.002
Effect of glucagon on intrapancreatic fat	0.251	0.152	0.099

Data on liver fat, HbA_1c_, fasting plasma glucose, fasting insulin, total amylin, C-peptide, total GLP-1, GIP, glucagon, ALT, AST, ALP, GGT and triacylglycerol were log-transformed

Single-mediator model with single-level data was used to investigate mediation. All mediation analyses were adjusted for age, sex, ethnicity and BMI

ALP, alkaline phosphatase; ALT, alanine aminotransferase; AST, aspartate aminotransferase; GGT, γ-glutamyl transpeptidase

**Table 3:** Indirect and direct effect estimates with bootstrap CIs

Effect	Estimated coefficient	95% CI	Proportion mediated (%)^a^
Direct effect on intrapancreatic fat	0.412	0.186, 0.639	-
Indirect effect through HbA_1c_	−0.007	−0.036, 0.014	-
Direct effect on intrapancreatic fat	0.468	0.254, 0.682	-
Indirect effect through fasting plasma glucose	0.032	0.001, 0.077	6.8
Direct effect on intrapancreatic fat	0.453	0.216, 0.690	-
Indirect effect through fasting insulin	0.020	−0.004, 0.063	-
Direct effect on intrapancreatic fat	0.407	0.153, 0.662	-
Indirect effect through triacylglycerol	0.099	0.033, 0.189	24.3
Direct effect on intrapancreatic fat	0.428	0.210, 0.646	-
Indirect effect through total cholesterol	0.009	−0.010, 0.037	-
Direct effect on intrapancreatic fat	0.459	0.242, 0.676	-
Indirect effect through HDL-cholesterol	0.082	0.027, 0.151	17.9
Direct effect on intrapancreatic fat	0.440	0.218, 0.662	-
Indirect effect through LDL-cholesterol	0.009	−0.019, 0.021	-
Direct effect on intrapancreatic fat	0.514	0.253, 0.776	-
Indirect effect through total amylin	0.008	−0.014, 0.043	-
Direct effect on intrapancreatic fat	0.496	0.231, 0.761	-
Indirect effect through C-peptide	0.015	−0.012, 0.052	-
Direct effect on intrapancreatic fat	0.594	0.327, 0.861	-
Indirect effect through total GLP-1	−0.012	−0.058, 0.023	-
Direct effect on intrapancreatic fat	0.578	0.308, 0.849	-
Indirect effect through GIP	0.003	−0.060, 0.066	-
Direct effect on intrapancreatic fat	0.514	0.253, 0.776	-
Indirect effect through glucagon	0.013	−0.015, 0.048	-
Direct effect on intrapancreatic fat	0.653	0.364, 0.942	-
Indirect effect through ALT	0.044	−0.012, 0.130	-
Direct effect on intrapancreatic fat	0.653	0.364, 0.942	-
Indirect effect through AST	0.005	−0.050, 0.061	-
Direct effect on intrapancreatic fat	0.653	0.364, 0.942	-
Indirect effect through ALP	−0.003	−0.076, 0.078	-
Direct effect on intrapancreatic fat	0.653	0.364, 0.942	-
Indirect effect through GGT	0.054	−0.001, 0.135	-

Data on liver fat, HbA_1c_, fasting plasma glucose, fasting insulin, total amylin, C-peptide, total GLP-1, GIP, glucagon, ALT, AST, ALP, GGT and triacylglycerol were log-transformed

The single-mediator model with single-level data was used. All the mediation analyses were adjusted for age, sex, ethnicity and BMI

^a^Proportion mediated represents an estimate of the extent to which the direct effect was accounted for by the pathway through the mediating variable. Significance of mediation was tested by computing bias-corrected bootstrap confidence intervals with 5000 repetitions

### Markers of glucose metabolism as mediators

Fasting plasma glucose and HbA_1c_ were considered as potential mediators in the association between liver fat and IPFD (Fig. 2). Both the exposure–mediator effect and the mediator–outcome effect were statistically significant for glucose but neither were significant for HbA_1c_ (Table 2). Based on the statistical significance of the indirect effect estimates, glucose was a mediator whereas HbA_1c_ was not (Fig. 3). Glucose mediated 6.8% of the association between liver fat and IPFD (Table 3).

### Incretins as mediators

GLP-1 and GIP were considered as potential mediators in the association between liver fat and IPFD (Fig. 2). While the exposure–mediator effect was not statistically significant for either GLP-1 or GIP, the mediator–outcome effect was statistically significant for GIP but not GLP-1 (Table 2). Based on the statistical significance of the indirect effect estimates, neither incretin was a mediator (Fig. 3).

### Pancreatic hormones and derivatives as mediators

Insulin, amylin, C-peptide and glucagon were considered as potential mediators in the association between liver fat and IPFD (Fig. 2). Neither the exposure–mediator effect nor the mediator–outcome effect was statistically significant for any of the studied pancreatic hormones (Table 2). Based on the statistical significance of the indirect effect estimates, none of the pancreatic hormones were mediators (Fig. 3).

### Liver enzymes as mediators

Alanine aminotransferase, aspartate aminotransferase, alkaline phosphatase and γ-glutamyl transpeptidase were considered as potential mediators in the association between liver fat and IPFD (Fig. 2). While the exposure–mediator effect was statistically significant for all the studied liver enzymes, the mediator–outcome effect was not statistically significant for any of them (Table 2). Based on the statistical significance of the indirect effect estimates, none of the liver enzymes were mediators (Fig. 3).

## Discussion

This study represents one of the largest MRI cohorts of people with measured IPFD in the literature. The 2008 ‘twin cycle’ hypothesis theorised the interconnectedness of IPFD and liver fat as a key factor influencing the development of type 2 diabetes as well as its remission [36]. The predictions of this hypothesis were subsequently confirmed in interventional studies of a low-energy diet: the Counterpoint, Counterbalance and DiRECT trials [37–39]. Further, a 2022 data-driven cluster analysis partitioned individuals without diabetes based on their abdominal fat distribution and then studied the longitudinal association of membership in the resulting clusters with incident type 2 diabetes [40]. The study showed that individuals with excess liver fat and those with excess IPFD (but not those with excess skeletal muscle fat deposition) are at remarkably similar (fourfold and 3.4-fold higher, respectively) risk of type 2 diabetes, further supporting the link between liver fat and IPFD [40]. The use of the path-analytic methodological framework and the large sample size of the present observational study enabled us to provide robust complementary evidence that supports the interconnectedness of IPFD and liver fat. We demonstrated, for the first time, that the influence of liver fat on IPFD can be partitioned into direct and indirect components using mediation analysis. Out of the 16 metabolic traits studied, three traits (triacylglycerol, HDL-cholesterol and glucose) met the established formal criteria for mediators. This held true irrespective of age, sex, ethnicity and BMI. Further, we were able to quantify the magnitude of the indirect effect that these circulating mediators have on the association between liver fat and intrapancreatic fat, ranging from 24.3% for triacylglycerol to 6.8% for glucose.

These three metabolic traits were previously identified as reasonably accurate biomarkers of IPFD in our 2017 meta-analysis of small observational studies [22]. Among the lipid metabolism-related metabolic traits identified, triacylglycerol and HDL-cholesterol had weighted correlation coefficients of 0.38 (95% CI 0.31, 0.46) and −0.33 (95% CI −0.35, −0.31), respectively, with IPFD [22]. They were investigated in seven studies (with 10.5% heterogeneity) and five studies (with 0% heterogeneity), respectively. When individuals with and without fatty pancreas were compared, triacylglycerol had a large positive standardised mean effect size (d+ = 0.49) whereas HDL-cholesterol had a medium negative standardised mean effect size (d+ = −0.32) [22]. In addition, glucose had a weighted *r* of 0.30 (95% CI 0.26, 0.33) with IPFD and was investigated in seven studies (with 0% heterogeneity) [22]. When individuals with and without fatty pancreas were compared, glucose had a medium positive standardised mean effect size (d+ = 0.36) [22]. However, the above correlations between IPFD and the three metabolic traits were investigated predominantly in individuals with obesity and/or diabetes but rarely in healthy non-obese individuals. The weighted mean correlation coefficients of IPFD were higher with HbA_1c_ than with glucose (*r*=0.39 vs *r*=0.30) in that meta-analysis. However, while the studies on glucose had 0% heterogeneity, those on HbA_1c_ had 77.8% heterogeneity. In addition, only six out of 17 studies included in the 2017 meta-analysis used MRI [6]. Most importantly, correlation is one of the simplest statistical analyses and it cannot provide insights into the extent to which potential causal variables influence IPFD.

The present study adds to the existing literature by delineating potentially causal mechanisms that are involved in deposition of fat in the pancreas. Of the three metabolic traits identified as mediators in the present study, triacylglycerol had the largest mediation effect on the association between liver fat and IPFD. Liver fat increased triacylglycerol by 18% of an SD. One SD increase in triacylglycerol changed IPFD by a factor of 0.61. The indirect effect of triacylglycerol on the association between liver fat and IPFD accounted for 24.3% of the direct effect. HDL-cholesterol had the second-largest mediation effect on the association between liver fat and IPFD. Liver fat decreased HDL-cholesterol by 10% of an SD. One SD decrease in HDL-cholesterol changed IPFD by a factor of 0.83. The indirect effect of HDL-cholesterol on the association of liver fat with IPFD accounted for 17.9% of the direct effect. Last, glucose had the smallest mediation effect on the association between liver fat and IPFD. Liver fat increased glucose by 3% of an SD. One SD increase in fasting plasma glucose changed IPFD by a factor of 1.14. The indirect effect of glucose on the association of liver fat and IPFD accounted for 6.8% of the direct effect. Based on the above findings, triacylglycerol, HDL-cholesterol and glucose can be conceptualised as the conduits through which deposition of fat in the liver affects the deposition of fat in the pancreas.

Several limitations are to be acknowledged. First, although the path-analytic methodological framework employed in the present study has been used previously [32, 33], it has not been applied to body composition and metabolic traits. IPFD encompasses multiple subcategories with potentially disparate causal factors that may not be part of the same mechanisms [6]. In addition, one could argue which direction cause is occurring in cross-sectional studies or whether a variable is a presumed causal consequence of the exposure (which would help to clearly distinguish between a mediator and a confounder). Genome-wide and Mendelian randomisation studies may help to provide further causal evidence. Second, although the mediation analysis enabled us to gain important mechanistic insights into the pathogenesis of IPFD, IPFD and liver fat were measured at a single time point. Prospective longitudinal cohort studies are warranted to demonstrate conclusively the causal relationship between liver fat and IPFD. Third, a single-mediator model was used in the present study. It is possible that multiple mediators affect one another, and that these mediators may act as confounders of the effects of other mediators [34]. Given that triacylglycerol and HDL-cholesterol may not be causally independent, caution is necessary when interpreting the estimates of their effects in the present study. Fourth, histological confirmation of fat in the pancreas (and liver) was not available. This is because biopsy of the pancreas is highly invasive and cannot be ethically performed in a large study. However, the present study used 3.0 T MRI, the gold standard for non-invasive quantification of fat in the pancreas. Further, a single scanner/image acquisition protocol was used for all study participants, ensuring consistency of the measurements. Fifth, all the metabolic traits were investigated in the fasted state only. It is conceivable that the indirect effects of postprandial plasma glucose [41, 42], lipid profile [43, 44] and incretins [42, 45, 46] on the association between liver fat and IPFD may differ from those in fasted state. Further mediation analyses may consider exploring metabolic traits in a postprandial state. Last, habitual dietary intake is known to affect both liver fat and IPFD [47–50] but was not investigated in the present study. The role of dietary intake in the association between liver fat and IPFD remains to be studied in the future.

In conclusion, out of the 16 metabolic traits (lipid panel, liver enzymes, pancreatic hormones, markers of glucose metabolism and incretins) investigated in the present study, three traits, namely triacylglycerol, HDL-cholesterol and glucose, were established to be mediators in the association between liver fat and IPFD. This calls for retiring the simplistic paradigm of a direct linear association between liver fat and IPFD. Targeting the circulating levels of triacylglycerol, HDL-cholesterol and/or glucose holds promise for reducing IPFD.

## Data Availability

The datasets generated and analysed during the present study are available from the corresponding author upon reasonable request.

## Abbreviations

GIP: Gastric inhibitory peptide
GLP-1: Glucagon-like peptide-1
IPFD: Intrapancreatic fat deposition
MRS: Magnetic resonance spectroscopy
TE: Echo time
TR: Repetition time
